# Microwave-Assisted Extraction of Secondary Metabolites Using Ethyl Lactate Green Solvent from *Ambrosia arborescens*: LC/ESI-MS/MS and Antioxidant Activity

**DOI:** 10.3390/plants13091213

**Published:** 2024-04-27

**Authors:** Evelyn Guillen, Hector Terrones, Teresa Cano de Terrones, Mario J. Simirgiotis, Jan Hájek, José Cheel, Beatriz Sepulveda, Carlos Areche

**Affiliations:** 1 Departamento de Química, Facultad de Ciencias Naturales y Formales, Universidad Nacional de San Agustín, Arequipa 68513, Peru; eguillenmen@unsa.edu.pe (E.G.); hterrones@unsa.edu.pe (H.T.); dcanof@unsa.edu.pe (T.C.d.T.); 2 Instituto de Farmacia, Facultad de Ciencias, Campus Isla Teja, Universidad Austral de Chile, Valdivia 5090000, Chile; mario.simirgiotis@uach.cl; 3 Laboratory of Algal Biotechnology, Centre ALGATECH, Institute of Microbiology of the Czech Academy of Sciences, Opatovický Mlýn, 379 81 Třeboň, Czech Republic; hajek@alga.cz (J.H.); jcheel@alga.cz (J.C.); 4 Departamento de Ciencias Químicas, Universidad Andrés Bello, Campus Viña del Mar, Quillota 980, Viña del Mar 2520000, Chile; bsepulveda@uc.cl; 5 Departamento de Química, Facultad de Ciencias, Universidad de Chile, Las Palmeras 3425, Nuñoa, Santiago 8320000, Chile

**Keywords:** alternative solvents, *Ambrosia arborescens*, ethyl lactate, green extraction, microwave, non-conventional technology

## Abstract

Alternative solvents are being tested as green solvents to replace the traditional organic solvents used in both academy and industry. Some of these are already available, such as ethyl lactate, cyrene, limonene, glycerol, and others. This alternative explores eco-friendly processes for extracting secondary metabolites from nature, thus increasing the number of unconventional extraction methods with lower environmental impact over conventional methods. In this context, the Peruvian *Ambrosia arborescens* was our model while exploring a microwave-assisted extraction (MAE) approach over maceration. The objective of this study was to perform a phytochemical study including UHPLC-ESI-MS/MS and the antioxidant activity of *Ambrosia arborescens*, using sustainable strategies by mixing both microwaves and ethyl lactate as a green solvent. The results showed that ethyl lactate/MAE (15.07%) achieved a higher extraction yield than methanol/maceration (12.6%). In the case of the isolation of psilostachyin, it was similar to ethyl lactate (0.44%) when compared to methanol (0.40%). Regarding UHPLC-ESI-MS/MS studies, the results were similar. Twenty-eight compounds were identified in the ethyl lactate/MAE and methanol/maceration extracts, except for the tentative identification of two additional amino acids (peaks 4 and 6) in the MeOH extract. In relation to the antioxidant assay, the activity of the ethyl lactate extract was a little higher than the methanol extract in terms of ORAC (715.38 ± 3.2) and DPPH (263.04 ± 2.8). This study on *A. arborescens* demonstrated that the unconventional techniques, such as MAE related to ethyl lactate, could replace maceration/MeOH for the extraction and isolation of metabolites from diverse sources. This finding showed the potential of unconventional methods with green solvents to provide eco-friendly methods based on green chemistry.

## 1. Introduction

*Ambrosia arborescens* Miller is an aromatic medicinal plant that belongs to the Asteraceae family. This species is common in Peru, Colombia, Ecuador, and Bolivia at altitudes between 2000 and 3500 m above sea level. It is commonly known as “marco” and its conservation status is common and abundant. Its leaves are used externally in traditional medicine for rheumatism and muscle pain [1]. Extraction of secondary metabolites from plants requires various isolation techniques because the plants’ metabolite content is generally low, and extraction techniques are often the bottleneck in natural product chemistry. These techniques can be divided into conventional and unconventional. The conventional techniques, which include maceration, percolation, and Soxhlet extraction, are the most widely used, and they are characterized by high cost, long extraction times, and the use of toxic organic solvents. Unconventional or modern techniques include ultrasound, microwave, enzyme extraction, supercritical fluid extraction, and high pressure, which are characterized by lower cost, shorter extraction time, and energy conservation [2]. 

Chemat et al. (2012) propose the following definition for green extraction of natural products: “Green extraction is based on the discovery and development of extraction processes that reduce energy consumption, allow the use of alternative solvents and renewable natural products, and ensure a safe and high-quality extract or product”. With this in mind, there are six principles for environmentally friendly extraction of natural products that all chemists should be aware of in order to follow an environmentally conscious path in natural product chemistry: 1. Innovate by selecting and using varieties of renewable plant resources. 2. Use alternative solvents, especially water or agro-solvents. 3. Reduce energy consumption through energy recovery and use of innovative technologies. 4. Generate byproducts instead of waste involving bio- and agro-refining industry. 5. Reduce operation units and promotion of safe, robust and controlled processes. 6. Aim for a non-denatured and biodegradable extract without impurities [3]. 

Alternative solvents have been tested as green solvents to replace the traditional organic solvents used in both in academy and industry. A green solvent should be biodegradable, recyclable, non-toxic, non-volatile, cheap, and readily available, such as ethyl lactate, cyrene, limonene, glycerol, deep eutectic solvents, and water [2]. Many natural product chemists base their research on considering the health of the planet. Thus, the implementation of ecopharmacognosy proposed by Cordell (2015) will be incorporated into the process of future natural product research [4]. It is known that the main ecological challenge in phytochemistry is the use of solvents due to the risk of environmental pollution [4,5]. This fact has opened up the search for more efficient processes in combination with more environmentally friendly solvents, whose negative impact is lower. In this sense, many pharmaceutical companies such as Pfizer, GlaxoSmithKline, AstraZeneca, and Sanofi have developed guidelines and new processes in this field. 

Based on this context, and continuing our research on green chemistry applied to plant natural products, we now report the results of a phytochemical study of green extracts of *Ambrosia arborescens* Miller obtained using the green solvent ethyl lactate and microwaves. Finally, all extracts were verified by UHPLC/ESI/MS/MS.

## 2. Results and Discussion

### 2.1. Extraction Using Conventional or Non-Conventional Techniques

Powdered samples of *A. arborescens* (50 g) were extracted with MeOH and ethyl lactate, using conventional or non-conventional techniques. Maceration was used in the first case and microwaves in the second. Extraction with methanol and ethyl lactate (MAE) yielded 12.5% and 15.0%, respectively. These results suggest that ethyl lactate could replace methanol as an extraction solvent despite its higher cost. Thus, these results suggest that the non-conventional technique based on MAE is better than maceration. 

### 2.2. Isolation

**Figure 1:**
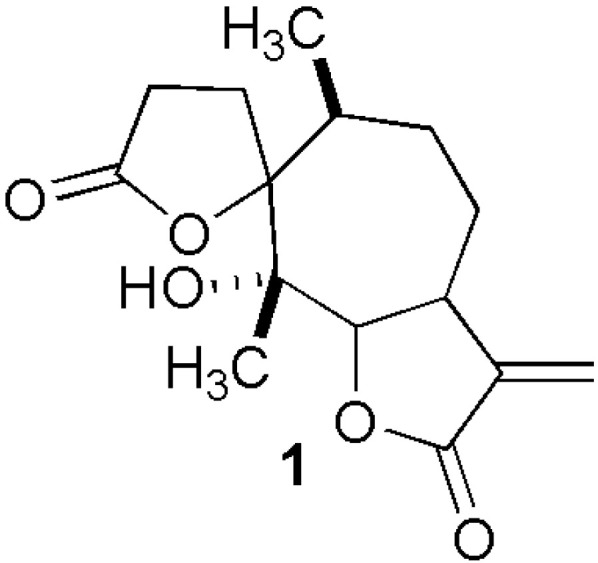
Chemical structure of psilostachyin.

To isolate the main compound, crude extracts (CM and NCM) obtained after solvent evaporation were fractionated by both flash column chromatography (SiO_2_) and CC (SiO_2_). Psilostachyin **1** was isolated from methanol (maceration) and ethyl lactate (MAE) extracts, in yields of 0.40% and 0.44%, respectively. Psilostachyin was identified based on spectroscopic analysis (^1^H-NMR). The chemical structure of psilostachyin is shown in Figure 1. This result shows that the isolation of psilostachyin with green solvents is comparable to that of methanol in terms of yield. On the other hand, the extraction of metabolites from plants usually requires the use of organic solvents such as hexane, dichloromethane, acetone, methanol, or their mixtures. Hexane is used for the extraction of lipophilic metabolites, and it is the first choice due to its low price. Methanol is preferred because of its polar compounds. However, hexane and methanol have negative effects on the environment and living organisms. An alternative to the use of organic solvents for extraction could be ethyl lactate and heptane or their mixtures [6,7].

### 2.3. Metabolomic Profiling Using UHPLC-ESI-MS/MS

The ethyl lactate and methanolic extracts were selected for metabolic profiling and comparative purposes, as shown in Table 1. All compounds were compared and identified with previously published LC/MS/MS data [8,9,10,11,12,13].

#### 2.3.1. Carbohydrates

Peak 1 was tentatively identified as sucrose, with a molecular ion at *m*/*z* 341.1042. It was found in both extracts [8].

#### 2.3.2. Simple Organic Acids

Peaks 2, 3, and 5 were identified as quinic acid (C_7_H_12_O_6_), malic acid (C_4_H_5_O_5_), and succinic acid (C_4_H_5_O_4_), with *m*/*z* 191.0613, 133.0130, and 117.0201, respectively [8,9]. All these compounds were found in both extracts.

#### 2.3.3. Nitrogen-Containing Compounds

Peak 4 and peak 6 were identified by HRMS and MS/MS, respectively. Fragments of amino acids were detected, including leucine-hexose (*m*/*z* 292.1420; 130.0875) or its isomer isoleucine-hexose and phenylalanine (*m*/*z* 164.0713; 147.0492) [8,9]. These compounds were found in the methanolic extract and not in the ethyl lactate extract. 

#### 2.3.4. Hydroxybenzoic Acid and Its Derivatives

Peaks 7, 8, and 18 showed a deprotonated molecule at *m*/*z* 315.0745 (C_13_H_15_O_9_), in almost all cases. In the MS/MS, ions at 153.0200 corresponding to dihydroxybenzoic acid fragments were observed, indicating the presence of dihydroxybenzoic acid hexosides. Vanillic acid hexoside was assigned to peak 9 based on the losses of the aglycone (*m*/*z* 167.0351). Galloyl dihexoside (peak 13) showed one molecular ion at *m*/*z* 493.1188 and its daughter ions at *m*/*z* 331.0668 (loss of hexoside) and *m*/*z* 169.0149 (loss of two hexosides) [8,9,14]. All these hydroxybenzoic acids were found in both methanol and ethyl lactate extracts.

#### 2.3.5. Hydroxycinnamic Acids

Numerous studies have demonstrated the antioxidant potential of extracts containing hydroxycinnamic acids [8,9,14]. Six compounds (peaks 10–12 and 14–16) were tentatively identified in both extracts studied. In this regard, peaks 10–12 with molecular ions at *m*/*z* 353.0881, 353.0880, and 367.1045 correspond to monocaffeoylquinic acid, monocaffeoylquinic acid I, and caffeoylquinic acid methyl ester, respectively. All of these peaks have an identical daughter ion at 191.0562 (quinic acid). Peak 14 was identified as dicaffeoylquinic acid with molecular ions at *m*/*z* 515.1220 and its MS/MS fragments (caffeoylquinic acid, quinic acid, and caffeic acid). The isomeric peaks 15–16 were assigned to chlorogenic acid hexoside based on their fragments at *m*/*z* 353.0880 and 191.0566 [8,9,14]. 

#### 2.3.6. Flavonoid Derivatives

Among flavonoids, two compounds known as quercetin methyl ether (peak 20) and tetrahydroxy dimethoxy flavone (peak 21) were identified in both extracts [8,9,14]. 

#### 2.3.7. Terpenoid Derivatives

Based on HRMS, MS/MS, and findings from Ambrosia phytochemistry [10,11,12,13], five compounds belonging to two sesquiterpenoid skeletons (ambrosanolide and oplopanone) were tentatively identified. Peak 19 was assigned to the sesquiterpene lactone dehydropsilostachyin (*m*/*z* 281.1408; C_15_H_21_O_5_). Peak 19 produced fragment ions at *m*/*z* 237.1512, corresponding to the loss of CO_2_ and then an additional CO_2_ (*m*/*z* 191.1558). The sesquiterpene lactone psilostachyin was tentatively assigned to peak 23 based on its daughter ions at *m*/*z* 265.1451 (loss of CO_2_). Psilostachyin was isolated, as shown above, and identified by NMR in both extracts. Peak 24 was tentatively identified as a psilostachyin derivative (*m*/*z* 263.1357; C_15_H_19_O_4_). Peaks 25 (*m*/*z* 251.1717; C_15_H_23_O_3_) and 26 (*m*/*z* 249.11560; C_15_H_21_O_3_) were tentatively assigned to oxooplopanone and hydroxyoxooplopanone sesquiterpenoids, respectively [10].

#### 2.3.8. Lipid Derivatives

Four compounds were detected in both extracts of *A. arborescens*, including azelaic acid (peak 17), trihydroxyoctadecadienoic acid (peak 22), and hydroxyoctadecatrienoic acids (peaks 27 and 28) [8,9,14].

### 2.4. Antioxidant Activity

Flavonoid content, total phenolics, and antioxidant activity were evaluated by the Folin–Ciocalteau method and the ferric trichloride method, while antioxidant activity was expressed as FRAP, ferric reducing antioxidant power, ORAC, oxygen radical absorbance, and DPPH bleaching capacity (Table 2). MeOH extraction by maceration (CM) showed FRAP ferrous reduction ability (711.43 ± 23.2 mg/g extract), followed by ethyl lactate extraction by MAE (NCM, 705.22 ± 15.42 mg Trolox/g extract). In terms of DPPH quenching capacity, the extract of NCM (263.04 ± 2.9) was more effective than that of CM (180.47 ± 2.6). These results are in agreement with the ORAC assay (Table 2). Our results are in agreement with those from antioxidant plants, but lower in relation to fruits [15,16]. 

As you can see in Table 2, the TPC of NCM (ethyl lactate/MAE) is almost twice as low as the TPC of CM (MeOH/maceration), while the TFC of NCM is 2.5 times lower than that of CM. In this sense, even though NCM uses a green solvent, it produces more yield than CM. These are general data and do not take into account the real extraction capacity of both methodologies. Moreover, the FRAP potency is similar between the two extracts, while ORAC capacity is higher for the NCM (715.38 ± 3.2) than for the CM (541.13 ± 4.8), with the same trend occurring in the case of the quenching of the DPPH radical (Table 2). Therefore, this means that the green NCM extract can differentiate between and extract more antioxidant compounds than the MeOH extract. In terms of yields, both extracts are comparable; however, the green extract was a little higher (15.0%), thus having more antioxidant power.

In relation to the phytochemistry and biological activities of *A. arborescens*, Hilario-Vargas et al. reported the acute toxicity of an aqueous extract of the leaves of *A. arborescens* Mill at a single dose of 300 and 2000 mg/kg in rats. The study showed that the aqueous extract had an LD_50_ greater than 2000 mg/kg, causing mild congestion in the liver and kidneys based on creatinine, alanine aminotransferase (ALT), aspartate aminotransferase (AST), and histopathological examinations [17]. Another work indicated that the sesquiterpene lactones coronopilin and damsin from Bolivian *A. arborescens* inhibited the expression of the pro-inflammatory cytokines IL-6 and MCP-1 via NF-κB inhibition, suggesting that they could be useful as anti-inflammatory agents for human skin [18]. In our case, psilostachyin and coronopilin were tentatively identified by LC/MS/MS. Psilostachyin was isolated from Peruvian *A. arborescens* as the main component, while damsin (13.4 mg/g) and coronopilin (12.3 mg/g) were detected in high concentrations in the Bolivian sample. These results indicate the presence of damsin in Bolivian samples, but not in Peruvian plants, which could be due to environmental conditions including the climatic and edaphic factors of these countries. In cancer research, the sesquiterpene lactones damsin, coronopilin, ambrosin, and dindol 01 inhibited cell proliferation by down-regulating cyclin-dependent kinase 2 at doses up to 5 µM. Moreover, these compounds inhibited tumor necrosis factor-α, and damsin and ambrosin decreased the subpopulation of cancer stem cells [19], while coronopilin inhibited leukemia cells with an IC_50_ ≤ 20 µM [20]. On the other hand, Solís-Quispe et al. (2022) identified one hundred and thirteen essential oil compounds, with sesquiterpenes and monoterpenes being the most important classes. Moreover, it was found that the essential oil exhibited a free radical scavenging effect, in addition to antioxidant activity, by inhibiting lipid peroxidation in the brain of rats [21]. It is known that the Ambrosia genus produces characteristic chemical skeletons such as pseudoguaianolides, eudesmanes, and oplopanones [10,11,12,13]. One of the first reports on Peruvian *A. arborescens* described the isolation of the sesquiterpenoids damsin, coronopilin, psilostachyin, and psilostachyin C [22]. On the other hand, De Leo et al. 2010 [10] reported the presence of sesquiterpenoids such as damsin, damsinic acid, 10α-hydroxydamsin, psilostachyin, psilostachyin C, 10α-hydroxy-11,13-dihydro-5-epi psilostachyin, volenol, 12-hydroxy-4(5), 11(13)-eudesmadien-15-al, 4(15)-eudesmene-1β,7α-diol, eudesm-11(13)-en-4β,9β-diol, dehydrocoronopilin, coronopilin, 13-hydroxy-4-oxo-7(11)-pseudoguaien-12,6-olide, 4β-hydroxypseudoguaian-12,6-olide-4-O-β-D-glucopyranoside, and 1α-hydroxy-7-oxo-iso-anhydrooplopanone in the leaves of *A. arborescens* from Ecuador. In addition, the two diterpenoids 15R,16-dihydroxy-3-oxoisopimar-9(11)-ene and 15S,16-dihydroxy-3-oxoisopimar-9(11)-ene were isolated. These results are of great importance for *Ambrosia* chemistry, as six new compounds were detected for the first time. 

From the point of view of green extraction techniques and green isolation, the use or substitution of toxic solvents was minimal. In our case, psilostachyin was isolated from a 50 g sample of Peruvian *A. arborescens* using ethyl lactate (green solvent) with mixtures of n-heptane (GRAS solvent), representing a new transition from classical to green isolation in natural product chemistry. Although the yields were comparable to those of methanol, this is the first report of the application of green chemistry to *A. arborescens*. 

The green extraction process is one important step for alternative phytochemical analysis in natural product chemistry. This process determines the isolation, identification, and composition of secondary metabolites prior to their assessment by analytical techniques such as LC-ESI/MS/MS. In recent years, a combination of unconventional techniques with green solvents has been used to promote a substantial reduction of toxic solvents in favor of producing higher quality extracts. A promising method includes MAE with ethyl lactate solvent; this extraction is simple, ecological, and low-energy and involves reduced time for extraction and low consumption of solvents for the extraction of compounds from natural sources. In this context, MAE has demonstrated itself to be an advantageous method for the extraction with the following influential factors: volume of the solvent, microwave power, extraction time, matrix, and temperature. All works reported in the literature include a combination of these factors to obtain caffeine and polyphenols from green tea; phenolic acids, flavonoid and its glycosides from *Hibiscus sabdariffa*; polyphenols and flavonoids from *Cynara scolymus*; pectins from orange peel; polyphenols in quercus shells; tannins and flavonoids from *Myrtus communis* leaves; polyphenols of stems, flowers, and seeds from *Ocimum basilicum*; and oleuropein from olive leaves [23,24].

In relation to the use of ethyl lactate as an alternative solvent, it shows a direct relationship with the green criterion, and it follows green chemistry principles. Ethyl lactate is biodegradable with an oral LD_50_ over 2000 mg/kg, with properties such as low surface tension, low vapor pressure, and high boiling point. Thus, it can replace organic solvents such as acetone, xylene, toluene, methyl ethyl ketone, or other toxic solvents for many applications including paints, cleaning of graffiti, composites, and adhesives, among others [25]. Therefore, the use of ethyl lactate is increasingly available in academic laboratories as alternative extracting agent to traditional solvents. In recent years, the use of ethyl lactate for extraction and separation has increased, showing the potential use of this solvent in natural product chemistry, such as for carotenoids and their congeners, tocopherol, sclareol, quercetin, rutin, caffeine, amino acids, and phenols from lichens [26,27,28,29,30,31,32,33].

The use of unconventional techniques, together with green solvents, could open new avenues for the extraction and isolation of secondary metabolites from plants, in order to provide eco-friendlier processes.

## 3. Materials and Methods

### 3.1. Plant Material

Specimens were collected in 2019 in Distrito de Chiguata, Arequipa, Peru (GPS coordinates: 16°01′28″ N, 71°01′25″ E) and identified as *Ambrosia arborescens* Miller by Prof. Fatima Caceres of San Agustin University, Arequipa, Peru. A voucher specimen (N° AMA-03032020) was delivered from the Herbarium of the Universidad de San Agustin.

### 3.2. Chemicals

TLC (Kieselgel 60 GF254, Merck, Darmstadt, Germany) was performed in heptane/EtOAc mixtures (9:1; 7:3 and 1:1 *v*/*v*), and spots were visualized by spraying plates with H_2_SO_4_-MeOH (5:95, *v*/*v*) and heating to 120 °C. Silica gel (Kieselgel 60, Merck 0.063–0.200 mm) and Sephadex (LH-20, Merck) were used for column chromatography (CC). Ethyl lactate was purchased from Sigma Aldrich (Saint Louis, MO, USA). Purified water (TOC < 5 µg/L) was obtained from a reverse osmosis system (Arium 126 61316-RO and Arium 611 UV unit (Sartorius, Goettingen, Germany)). Methanol (HPLC grade) and formic acid (for mass spectrometry, puriss. p.a.) were purchased from J.T. Baker (Phillipsburg, NJ, USA). Folin–Ciocalteu (FC) reagent, 2,2-diphenyl-1-picrylhydrazyl (DPPH), ferric chloride hexahydrate, 2,4,6-tris(2-pyridyl)-s-triazine, Trolox, quercetin, gallic acid, di-methyl sulfoxide (DMSO), and trichloroacetic acid were purchased from Merck (Darmstadt, Germany). 

### 3.3. Maceration Extraction

The powdered sample of *Ambrosia arborescens* (200 mg) was added to 10 mL of methanol at room temperature with constant stirring (200 rpm) and left for 24 h. After centrifugation (9000× *g*, 30 min), the methanolic solution was evaporated in vacuum to yield 25.07 ± 0.4 mg of a dark extract (12.6 ± 0.21%). Extractions were performed in triplicate following previous work [34].

### 3.4. Microwave-Assisted Extraction (MAE)

The powdered samples of *A. arborescens* (200 mg) were placed in 10 mL of ethyl lactate. They were then put in a microwave reactor (Anton Parr, Buchs, Switzerland) with adjustable parameters (100 °C, 30 min, 15 W). After centrifugation at 9000× *g* for 30 min, the supernatant was evaporated under vacuum to yield a dark gummy extract (29.93 ± 1.2 mg; 15.0 ± 0.62%). Extractions were performed in triplicate following previous work [34].

### 3.5. Extraction and Isolation 

Maceration and MAE in green solvents were used to prepare extracts, along with conventional organic solvents to fractionate and purify the main compound for comparative purposes. 

#### 3.5.1. Conventional Methods

Dried and powdered material of *A. arborescens* (50 g) was macerated with methanol (3 times, 1.0 L, 3 days/extraction), yielding a methanolic extract (CM; 6 g). This extract (5 g) was subjected to flash chromatography on silica gel (63–200 µm, 60 g, column length 15 cm, i.d. 10 cm) and eluted with n-hexane/EtOAc mixtures (1 L each) of increasing polarity (7:3, 4:6, 0:1; *v*/*v*) to yield three fractions (CM 1–3). Fraction CM-1 (1 g, n-hexane/EtOAc 7:3) was submitted to CC and eluted with EtOAc-n-hexane (0:1, 0.5:9.5, 1:9, 1.5:8.5, 2:8, 3:7 *v*/*v*) and combined into two fractions (ACM-1A and ACM-1B) after TLC comparison. CC (silica gel 63–200 µm, 70 g) on fraction ACM-1A (0.7 g), eluted with n-hexane-EtOAc mixtures (0–30% EtOAc) resulted in the isolation of the main compound **1**, known as psilostachyin (150 mg) [35]. The fraction CM-2 (0.8 g, n-hexane/EtOAc 4:6) was purified by CC using n-hexane-EtOAc mixtures (20–60% EtOAc) as the mobile phase, allowing compound **1** (50 mg) to be separated again.

#### 3.5.2. Non-Conventional Methods

MAE was used together with green solvents to obtain the green extracts. Fractionation and purification of *A. arborescens* (50 g) after microwave extraction was performed as described above. Subsequently, the ethyl lactate solution was concentrated under reduced pressure, yielding a green extract (NCM; 7.5 g). This extract (5.0 g) was subjected to CC on silica gel (63–200 µm, 60 g, column length 15 cm, i.d. 10 cm) and eluted with n-heptane/ethyl lactate mixtures (1 L each) of increasing polarity (7:3, 4:6; 0:1; *v*/*v*) to produce three fractions (NCM 1–3). Fraction NCM-1 (1 g, n-heptane/EL 7:3) was submitted to CC and eluted with n-heptane/ethyl lactate (0:1, 0.5:9.5, 1:9, 1.5:8.5, 2:8, 3:7 *v*/*v*) and combined according to their similarity after TLC comparison, yielding psilostachyin as the main compound (160 mg). Fraction NCM-2 (0.8 g, n-heptane/EL 4:6) was purified under the same conditions as CM-2, where the compound **1** (60 mg) was re-isolated.

### 3.6. UHPLC-APCI-HRMS/MS Instrument 

The chemical identity of the target compounds was determined through a Dionex UltiMate 3000 HPLC system (Thermo Scientific, Waltham, MA, USA) connected to a high-resolution tandem mass spectrometry (HRMS/MS) detector with atmospheric pressure chemical ionization (APCI) source (Impact HD mass spectrometer Bruker, Billerica, MA, USA) (HPLC-APCI-HRMS). Some 2 mg of extract (obtained of the point 3.3 and 3.4) was dissolved in methanol (2 mL) and then filtered (PTFE filter), and finally 10 µL was injected into the instrument, as previously described [34,36]. 

#### HPLC and MS Parameters 

HPLC was performed using a C18 column (Accucore, 150 mm × 4.6 mm ID, 2.5 µm, Thermo Fisher Scientific, Bremen, Germany) at 25 °C. Detection wavelengths were 254, 280, 330, and 354 nm, and PDA from 200 to 800 nm for peak identification. The mobile phase was 0.1% formic acid aqueous solution (A) and 0.1% formic acid in acetonitrile (B). The gradient program (time (min), % B) was: (0.00, 12); (5.00, 12); (10.00, 20); (15.00, 40); (20.00, 40); (25.00, 70); (35.00, 12) and 15 min for column equilibration before each injection. The flow rate was 1.00 mL min^−1^, and the injection volume was 10 µL. The extracts and standards dissolved in methanol were kept at 10 °C during storage in the auto sampler. The parameters of the Impact HD mass spectrometer were set as previously described [8,34,36].

### 3.7. Antioxidant and Content of Phenolic Assays 

#### 3.7.1. Polyphenol and Flavonoid Contents

The determination of total phenolic compounds (TPC) was performed following protocols previously described [8,36]. Approximately 12 μL of the extract and 168 μL of 1% Folin–Ciocalteu reagent (Merck) were added to a well of a microplate reader. The mixture was allowed to react for 5 min, and then 120 μL of 10% Na_2_CO_3_ was added. The mixture was incubated at room temperature for 30 min in darkness. Then, the absorbance was measured at 765 nm using a UV-visible multiplate reader (Synergy HTX, Billerica, MA, USA). The content of phenolic compounds was then expressed as micromoles of gallic acid per gram of dry weight (μmol GAE/g extract). The AlCl_3_ method was used to determine the total flavonoid content. For this assay, 30 μL of sample (2 mg/mL) was added to 159 µL of 5% NaNO_2_. After a 5-min rest period, 18 μL of 10% AlCl_3_ was added to the mixture. At the sixth minute, 18 μL of 1 M NaOH was added, and the absorbance was measured at 510 nm using a UV-visible multiplate reader (Synergy HTX, Billerica, MA, USA). The flavonoid content (TFC) was calculated using a quercetin standard calibration curve (25–150 ppm). Results were expressed as quercetin micromoles per gram of dry sample (μmol Q/g dry weight).

#### 3.7.2. DPPH Test

The DPPH radical was measured by the decolorization method [8,36]. Briefly, 9 μL of green or methanol extract (2 mg/mL) and 341 μL methanolic DPPH solution (400 μM) were adjusted with methanol to an absorbance of 1.10 ± 0.02 at 517 nm. The mixture was mixed and left to react in the dark at room temperature for 20 min, after which the time absorbance at 517 nm was measured. The percentage of discoloration of the DPPH radical was determined by measuring the change in absorbance at 517 nm, and the values obtained were converted to percentage inhibition of the DPPH moiety. A curve was plotted with different dilutions of the extract, and the results were expressed as IC_50_ in µg/mL.

#### 3.7.3. Ferric Reducing Antioxidant Power Test (FRAP)

The methodology was performed as published with slight modifications [8,36]. Briefly, 10 μL of the solubilized extracts (2 mg/mL) were mixed with 290 μL of the FRAP solution, mixed in the wells of the microplate, and left to react in the dark at room temperature for 5 min. Absorbance measurement of the stained Fe-TPTZ complex was performed at 595 nm. The absorbance data were fitted to the Trolox standard curve equation (μmol/L). Results were calculated as Trolox equivalents (TE), in mg Trolox per gram dry weight (mg Trolox/g dry weight).

#### 3.7.4. Oxygen Radical Absorbance Capacity (ORAC) Test

ORAC activity was determined according to the method previously described [8,36]. Fluorescein (3′,6′-dihydroxyspiro [2-benzofuran-3,9′-xanthene]-1-one) was prepared as stock solution (4 μM) in 75 mM phosphate buffer pH 7.4, stored at 4 °C, and used as fluorescent probe. AAPH reagent (203.4 mg) was freshly prepared in 15 mL of 75 mM phosphate buffer and used to generate the peroxyl radical. Trolox was used as an internal standard. In all experiments, 150 μL of fluorescein was added to each well. The blank wells received 25 μL of phosphate buffer, the standards received 25 μL of Trolox dilution, and the samples received 25 μL of sample. Excitation was monitored at 485 nm and emission at 528 nm with a band pass of 20 nm on an HTX Multi-Mode Microplate Reader. Results were determined by a quadratic regression equation (Trolox/sample vs. fluorescence decay curves) and expressed as μM Trolox equivalents per 100 g of dry plant.

### 3.8. Statistical Analysis

Results were reported as the mean ± S.D. In all experiments, statistical differences between samples and controls were checked by one-way analysis of variance (ANOVA) followed by Dunnett’s test. The significance level was set at *p* < 0.01. All statistical analyses were executed using GraphPad Prism 6 for Windows.

## 4. Conclusions

MAE was both used in combination with ethyl lactate to produce extracts and compared with a conventional method such as maceration to isolate and purify the main component of the Peruvian *A. arborescens*. Our study showed that the isolation of psilostachyin by MAE/ethyl lactate was comparable to maceration/methanol, based on the yield obtained (0.40–0.44%). In the metabolomic profile by UHPLC/ESI/MS/MS, the ethyl lactate and methanol extracts were similar in terms of secondary metabolites (in all, twenty-eight), except for the identification of two amino acids (peaks 4 and 6) in the MeOH extract. Finally, in relation to the antioxidant assay, the activity of the ethyl lactate extract was a little higher than the methanol extract in terms of ORAC (715.38 ± 3.2) and DPPH (263.04 ± 2.8). These unconventional techniques, together with the use of green solvents, could replace toxic solvents for the extraction and isolation of secondary metabolites from plants and provide more sustainable methods based on green chemistry.

## Figures and Tables

**Table 1:** LC-ESI-MS/MS identification of secondary metabolites from Peruvian *A. arborescens*.

Peak	T_R_ (min)	Compounds	Formula Molecular	Theoretical Mass	Experimental Mass	MS Ions (*m/z*)	Solvent Maceration	Solvent MAE
1	3.33	Sucrose	C_15_H_17_O_9_	341.1031	341.1042	179.0569	MeOH	EL
2	3.47	Quinic acid	C_7_H_12_O_6_	191.0561	191.0563	---	MeOH	EL
3	4.78	Malic acid	C_4_H_5_O_5_	133.0149	133.0139	---	MeOH	EL
4	5.65	Leucine/isoleucine-hexose	C_12_H_22_NO_7_	292.1402	292.1420	202.1085, 130.0875	MeOH	-
5	6.54	Succinic acid	C_4_H_5_O_4_	117.0193	117.0201	---	MeOH	EL
6	10.10	Phenylalanine	C_9_H_10_NO_2_	164.0717	164.0713	147.0492	MeOH	-
7	10.24	Dihydroxybenzoic acid hexoside	C_13_H_15_O_9_	315.0735	315.0745	153.0200	MeOH	EL
8	11.66	Dihydroxybenzoic acid hexoside I	C_13_H_15_O_9_	315.0734	315.0740	---	MeOH	EL
9	12.47	Vanillic acid hexoside	C_14_H_17_O_9_	329.0878	329.0886	167.0351	MeOH	EL
10	14.07	Monocaffeoylquinic acid	C_16_H_17_O_9_	353.0878	353.0881	191.0563	MeOH	EL
11	14.61	Monocaffeoylquinic acid I	C_16_H_17_O_9_	353.0878	353.0880	191.0562	MeOH	EL
12	15.14	Caffeoylquinic acid methyl ester	C_17_H_20_O_9_	367.1035	367.1045	191.0565	MeOH	EL
13	15.54	Galloyl dihexoside	C_19_H_26_O_15_	493.1199	493.1188	331.0668 169.0149	MeOH	EL
14	15.81	Dicaffeoylquinic acid	C_25_H_23_O_12_	515.1195	515.1220	353.0871 191.0566 179.0350	MeOH	EL
15	16.35	Chlorogenic acid hexoside	C_22_H_27_O_14_	515.1418	515.1428	353.0880 191.0560	MeOH	EL
16	17.02	Chlorogenic acid hexoside I	C_22_H_27_O_14_	515.1418	515.1428	353.0881 191.0566	MeOH	EL
17	18.76	Nonanedioic acid (azelaic acid)	C_9_H_16_O_4_	187.0976	187.0984	---	MeOH	EL
18	19.83	Dihydroxybenzoic acid hexoside II	C_13_H_15_O_9_	315.0735	315.0739	---	MeOH	EL
19	21.30	Dehydropsilostachyin	C_15_H_21_O_5_	281.1389	281.1408	191.1558 237.1512 253.1829 265.1447	MeOH	EL
20	21.83	Quercetin methyl ether	C_16_H_11_O_7_	315.0510	315.0523	---	MeOH	EL
21	22.37	Tetrahydroxy dimethoxy flavone	C_17_H_14_O_8_	345.0616	345.0629	---	MeOH	EL
22	22.64	Trihydroxyoctadecadienoic acid	C_18_H_31_O_5_	327.2177	327.2189	---	MeOH	EL
23	23.17	Psilostachyin	C_15_H_19_O_5_	279.1238	-	265.1451	MeOH	EL
24	23.98	Psilostachyin derivative or coronopilin	C_15_H_19_O_4_	263.1289	263.1357	---	MeOH	EL
25	24.64	Oxo-oplopanone	C_15_H_23_O_3_	251.1647	251.1717	---	MeOH	EL
26	25.45	Hydroxy-oxo-oplopanone	C_15_H_21_O_3_	249.1496	249.1560	---		
27	29.06	Hydroxyoctadecatrienoic acid	C_18_H_29_O_3_	293.2117	293.2198	---	MeOH	EL
28	29.46	Hydroxyoctadecatrienoic acid I	C_18_H_29_O_3_	293.2117	293.2198	---	MeOH	EL

**Table 2:** Total phenolic content, total flavonoid content, and antioxidant activity (FRAP, ferric reducing antioxidant power; ORAC, oxygen radical absorbance capacity; and DPPH bleaching capacity) of different extractions from *Ambrosia*.

Sample	TPC	TFC	FRAP *	ORAC *	DPPH *
NCM (ethyl lactate, MAE)	5.541 ± 0.54 ^a^	3.84 ± 0.43 ^b^	705.22 ± 15.42 ^c^	715.38 ± 3.2 ^d^	263.04 ± 2.8 ^e^
CM (MeOH, maceration)	9.581 ± 0.62	6.32 ± 0.44 ^g^	711.43 ± 23.2 ^c^	541.13 ± 4.8	180.47 ± 2.6

All values are expressed as means ± SD (n = 3). * micrograms per gram of plant. Values in the same column marked with the same letter are not statistically different (*p* < 0.05).

## Data Availability

The raw data that support the findings of this study are available on request from the authors.

## References

[1] De la Cruz H., Vilcapoma G., Zevallos P.A. (2007). Ethnobotanical study of medicinal plants used by the Andean people of Canta, Lima, Peru. J. Ethnopharmacol..

[2] Zullaikah S., Rachmaniah O., Utomo A.T., Niawanti H., Ju Y.H., Saleh H.M., Koller M. (2018). Green Separation of Bioactive Natural Products Using Liquefied Mixture of Solids. Green Chemistry.

[3] Chemat F., Abert Vian M., Cravotto G. (2012). Green Extraction of Natural Products: Concept and Principles. Int. J. Mol. Sci..

[4] Cordell G.A. (2015). Ecopharmacognosy and the responsibilities of natural product research to sustainability. Phytochem. Lett..

[5] Funari C.S., Rinaldo D., Bolzani V.S., Verpoorte R. (2023). Reaction of the phytochemistry community to Green chemistry: Insights obtained since 1990. J. Nat. Prod..

[6] Chemat F., Abert-Vian M., Fabiano-Tixier A.S., Strube J., Uhlenbrock L., Gunjevic V., Cravotto G. (2019). Green extraction of natural products. Origins, current status, and future challenges. TrAC Trends Anal. Chem..

[7] Pereira C.S.M., Silva V.M.T.M., Rodrigues A.E. (2011). Ethyl lactate as a solvent: Properties, applications and production processes—A review. Green Chem..

[8] Guerrero-Castillo P., Reyes S., Robles J., Simirgiotis M.J., Sepulveda B., Fernandez-Burgos R., Areche C. (2019). Biological activity and chemical characterization of *Pouteria lúcuma* sedes: A possible use of an agricultural waste. Waste Manag..

[9] Abu-Reidah I.M., Contreras M.M., Arraez-Roman D., Segura-Carretero A., Fernandez-Gutierrez A. (2013). Reversed-phase ultra-high-performance liquid chromatography coupled to electrospray ionization-quadrupole-time-of-flight mass spectrometry as a powerful tool for metabolomic profiling of vegetables: *Lactuca sativa* as an example of its application. J. Chromatogr. A.

[10] De Leo M., Vera Saltos M.B., Naranjo Puente B.F., De Tommasi N., Braca A. (2010). Sesquiterpenes and diterpenes from *Ambrosia arborescens*. Phytochemistry.

[11] Ata A., Diduck C., Udenigwe C.C., Zahid S., Decken A. (2007). New chemical constituents of *Ambrosia spilostachya*. Arkivoc.

[12] Oberti J.C., Silva G.L., Sosa V.E., Kulanthaivel P., Herz W. (1986). Ambrosanolides and secoambrosanolides from *Ambrosia tenuifolia*. Phytochemistry.

[13] Abdel-Salam N.A., Mahmoud Z.F., Ziesche J., Jakupovic J. (1984). Sesquiterpene lactones from *Ambrosia marítima* (Damssissa). Phytochemistry.

[14] Abu-Reidah I.M., Arraez-Roman D., Segura-Carretero A., Fernandez-Gutierrez A. (2013). Extensive characterization of bioactive phenolic constituents from globe artichoke (*Cynara scolymus* L.) by HPLC-DAD-ESI-QTOF-MS. Food Chem..

[15] Genskowsky E., Puente L.A., Pérez-Álvarez J.A., Fernández-López J., Muñoz L.A., Viuda-Martos M. (2016). Determination of polyphenolic profile, antioxidant activity and antibacterial properties of maqui [*Aristotelia chilensis* (Molina) Stuntz] a Chilean blackberry. J. Sci. Food Agric..

[16] Ramos L.C., Palacios J., Barrientos R.E., Gómez J., Castagnini J.M., Barba F.J., Tapia A., Paredes A., Cifuentes F., Simirgiotis M.J. (2023). UHPLC-MS Phenolic Fingerprinting, Aorta Endothelium Relaxation Effect, Antioxidant, and Enzyme Inhibition Activities of *Azara dentata* Ruiz & Pav Berries. Foods.

[17] Silva-Correa C.R., Villarreal-La Torre V.E., González-Siccha A.D., Cruzado-Razco J.L., Gonzalez-Blas M.V., Sagastegui-Guarniz W.A., Calderon-Peña A.A., Aspajo-Villalaz C.L., Hilario-Vargas J. (2022). Acute toxicity of aqueous extract of *Ambrosia arborescens* Mill. on biochemical and histopathological parameters in rats. Toxicol. Res..

[18] Svensson D., Lozano M., Almanza G.R., Nilsson B.O., Sterner O., Villagomez R. (2018). Sesquiterpene lactones from *Ambrosia arborescens* Mill. Inhibit pro-inflammatory cytokine expression and modulate NF-Kb signaling in human skin cells. Phytomedicine.

[19] Sotillo W.S., Villagomez R., Smiljanic S., Huang X., Malakpour A., Kempengren S., Rodrigo G., Almanza G., Sterner O., Oredsson S. (2017). Anti-cancer stem cell activity of a sesquiterpene lactone isolated from *Ambrosia arborescens* and of a synthetic derivative. PLoS ONE.

[20] Cotugno R., Fortunato R., Santoro A., Gallotta D., Braca A., De Tommasi N., Belisario M.A. (2012). Effect of sesquiterpene lactone coronopilin on leukaemia cell population growth cell type-specific induction of apoptosis and mitotic catastrophe. Cell Prolif..

[21] Solís-Quispe L., Pino J.A., Marín-Villa J.Z., Tomaylla-Cruz C., Solís-Quispe J.A., Aragón-Alencastre L.J., Hernández I., Cuellar C., Rodeiro I., Fernández M.D. (2022). Chemical composition and antioxidant activity of *Ambrosia arborescens* Miller leaf essential oil from Peruvian Andes. J. Essent. Oil Res..

[22] Herz W., Anderson G., Gibaja S., Raulais D. (1969). Sesquiterpene lactones of some *Ambrosia* species. Phytochemistry.

[23] Da Silva R.F., Carneiro C.N., De Souza C.B., Gomez F.J.V., Espino M., Boiteux J., Fernandez M.A., Silva M.F., Dias F.S. (2022). Sustainable extraction bioactive compounds procedures in medicinal plants based on the principles of green analytical chemistry: A review. Microchem. J..

[24] Majid I., Khan S., Aladel A., Dar A.H., Adnan M., Khan M.I., Awadelkareem A.M., Ashraf S.A. (2023). Recent insights into green extraction techniques as efficient methods for the extraction of bioactive components and essential oils from foods. CyTA-J. Food.

[25] Calvo-Flores F.G., Monteagudo-Arrebola M.J., Dobado J.A., Isac-Garcia J. (2018). Green and Bio-Based Solvents. Top. Curr. Chem. (Z).

[26] Ishida B., Chapman M.H. (2009). Carotenoid extraction from plants using a novel, enviromentally friendly solvent. J. Agric. Food Chem..

[27] Vicente G., Paiva A., Fornari T., Najdanovic-Visak V. (2011). Liquid-liquid equilibria for separation of tocopherol from olive oil using ethyl lactate. Chem. Eng. J..

[28] Tombokan X.C., Aguda R.M., Danehower D.A., Kilpatrick P.K., Carbonell R.G. (2008). Three-component phase behavior of the sclareol-ethyl lactate-carbon dioxide system for GAS applications. J. Supercrit. Fluids..

[29] Velho P., Requejo P.F., Gomez E., Macedo E.A. (2020). Novel ethyl lactate based ATPS for the purification of rutin and quercetin. Sep. Purif. Technol..

[30] Kua Y.L., Gan S., Morris A., Ng H.K. (2016). Ethyl lactate as a potential green solvent to extract hydrophilic (polar) and lipophilic (non-polar) phytonutrients simultaneously from fruit and vegetable by-products. Sustain. Chem. Pharm..

[31] Kamalanathan I., Canal L., Hegarty J., Najdanovic-Visak V. (2018). Partitioning of amino acids in the novel biphasic systems based on environmentally friendly ethyl lactate. Fluid Phase Equilibr..

[32] Rajkowska K., Simińska D., Kunicka-Styczyńska A. (2022). Bioactivities and Microbial Quality of *Lycium* Fruits (Goji) Extracts Derived by Various Solvents and Green Extraction Methods. Molecules.

[33] Sepulveda B., Benites D., Albornoz L., Simirgiotis M., Castro O., Garcia-Beltran O., Areche C. (2023). Green ultrasound-assisted extraction of lichen substances from *Hypotrachyna cirrhata*. Ethyl lactate, a better extracting agent tan metanol toxic organic solvent?. Nat. Prod. Res..

[34] Calla-Quispe E., Robles J., Areche C., Sepulveda B. (2020). Are Ionic Liquids Better Extracting Agents Than Toxic Volatile Organic Solvents? A Combination of Ionic Liquids, Microwave and LC/MS/MS, Applied to the Lichen *Stereocaulon glareosum*. Front. Chem..

[35] Borges del Castillo J., Manresa-Ferrero M.T., Rodriguez-Luis F., Vasquez-Bueno P., Joseph-Nathan P. (1981). ^13^C NMR study of psilostachyinolides from some *Ambrosia* species. Org. Magn. Reson..

[36] Areche C., Hernandez M., Cano T., Ticona J., Cortes C., Simirgiotis M., Caceres F., Borquez J., Echeverria J., Sepulveda B. (2020). *Corryocactus brevistylus* (K. Schum. ex Vaupel) Britton & Rose (Cactaceae): Antioxidant, Gastroprotective Effects, and Metabolomic Profiling by Ultrahigh-Pressure Liquid Chromatography and Electrospray High Resolution Orbitrap Tandem Mass Spectrometry. Front. Pharmacol..

